# Acute Effects of Myofascial Release on Foot Mobility and Performance in Basketball Players with Hypomobile Feet—A Randomized Controlled Trial

**DOI:** 10.3390/life14111404

**Published:** 2024-10-31

**Authors:** Nihat Sarıalioğlu

**Affiliations:** Faculty of Sports Sciences, Giresun University, Ahmet Taner Kışlalı Street, 28200 Giresun, Turkey; nihat.sarialioglu@giresun.edu.tr

**Keywords:** basketball, foot deformities, hypomobility, myofascial release

## Abstract

Hypomobility in the foot structure causes functional limitations. These functional limitations affect sportive performance negatively, especially in basketball, where dynamic movements such as jumping and sudden changes in direction are very intense. In this context, reducing hypomobility is important in terms of eliminating performance disorders caused by hypomobility. The purpose of this study was to investigate the acute effects of myofascial release on foot mobility and sportive performance in basketball players with hypomobile feet. This study was designed as randomized controlled experimental research. Twenty-four male basketball players (23.46 ± 2.81 years) participated in this study. The athletes were first subjected to foot mobility, balance and vertical jump tests. Then, the participants were divided into two groups: myofascial release (MR) and placebo myofascial release (PMR). Myofascial release (Graston technique) was applied to the MR group, and placebo myofascial release was applied to the PMR group. After application, foot mobility and performance measurements were performed again, and the results were analyzed. It was observed that there was a significant difference in right foot mobility (d = 4), left foot mobility (d = 6), vertical jump (d = 1.13) and dynamic balance (d = 1.03) parameters after application in the MR group. It was also determined that the acute effects of the intervention on foot mobility lasted for at least two hours (*p* < 0.001). There was no change in any parameter after application in the PMR group (*p* > 0.05). The results of this study show that a single session of myofascial release applied to the plantar sole temporarily increased foot mobility and improved vertical jump and dynamic balance performance in basketball players with chronic hypomobility.

## 1. Introduction

Hypomobile foot is a mechanical foot problem in which functional pronation cannot be achieved due to the lack of movement of the structures that make up the foot complex [1]. Foot mobility is influenced by the arch architecture of the foot, intrinsic muscles and the elastic components of the plantar fascia [2,3]. Disruption to any of these components of the foot can impair the mobility of the foot. In particular, in sports such as basketball, where jumping activity is a basic requirement, shortening of the intrinsic muscle tendons of the foot and hardening of the plantar fascia and arch structure may occur due to overload, various traumas, incorrect training models and incorrect shoe use, causing the foot to become hypomobile [4,5,6].

Hypomobility-induced mechanical disturbances in the foot structure cause limitations in ideal function, an inability to distribute weight evenly on the surface contact area and impairments in somatosensory input. This affects the proximal links in the kinetic chain and thus athletic performance [7,8]. Basketball is a sport in which limitations to the function of the foot can directly affect performance. This is because basketball requires advanced jumping and balance skills for high athletic performance [9,10]. These skills require a functional foot structure and proper foot mechanics. A key component of proper foot mechanics is sufficient foot mobility. Optimal movement ability in the foot segments ensures efficient load transfer to the ground during pressure moments to keep the body balanced and provides maximum force transmission from the ground during jumping. For these reasons, proper foot mechanics are also important for the successful execution of fast and sudden movements in high intensity exercise in coordination with jumping ability [11,12,13].

Hypomobility can be treated with various exercise models, surgical interventions, orthoses and myofascial release techniques. Myofascial release techniques are commonly used to restore ideal length and improve function in the myofascial complex [14,15,16]. When previous research is examined, it is seen that therapeutic approaches such as myofascial release applied to the plantar sole of the foot are mostly focused on pathological conditions such as plantar fasciitis, post-training pain reduction and anatomical disorders such as flat feet [17,18]. However, there is no comprehensive research on the effects of measures taken against inadequate foot mobility on physical performance. Considering the gaps in the literature and the effects of foot morphology on performance, it is important to reveal the effects of foot mobility on physical demands in order to contribute to taking the necessary measures to achieving the desired sportive performance in athletes. Therefore, the aim of this study was to investigate the acute effects of myofascial release on foot mobility and sportive performance in basketball players with hypomobile feet.

## 2. Materials and Methods

### 2.1. Study Design

This study was designed as randomized controlled experimental research. The CONSORT reporting guide was used in the research design [19]. All applications and measurements in the research were carried out at the Giresun University of Sports Sciences Performance laboratory. The ethical approval for the research was approved by the Giresun University Social Sciences, Science and Engineering Sciences Research Ethics Committee with the decision dated 11 January 2023 and numbered 01/07, and the research was conducted in accordance with the Declaration of Helsinki.

### 2.2. Participants

Twenty-four male basketball players with a mean age of 23.46 ± 2.81 years participated in this study voluntarily. The minimum sample size to be assigned to the groups was estimated using G*Power (version 3.1.9.2) software. The sample size estimation was based on the results of a reference article that evaluated the acute effects of myofascial release on lower-extremity joint range of motion using weight-bearing lunge test (WBLT) values [20]. The two-tailed analysis indicated that, for this study, a statistical significance level of α = 0.05, a power = 0.85, and an effect size of f = 1.02 were sufficient, leading to a minimum requirement of 11 participants per group, totaling 22 participants. To account for potential enrollment issues, an extra participant was added to each group, resulting in a total of 24 participants.

Male basketball players who have hypomobility in at least one foot, have been actively licensed for the last five years, and have participated in training at least three times a week (excluding rest periods in macrocycles) were included in the study. Participants who had experienced a serious foot or ankle injury within the last year, those who had engaged in high-intensity sport activities in the last 24 h, individuals who had used pharmacological products referred to as muscle relaxants and their derivatives within the last 24 h, and those who did not meet the inclusion criteria were excluded from the study. All demographic data of the sample are shown in Table 1.

### 2.3. Procedures

**Figure 1:**
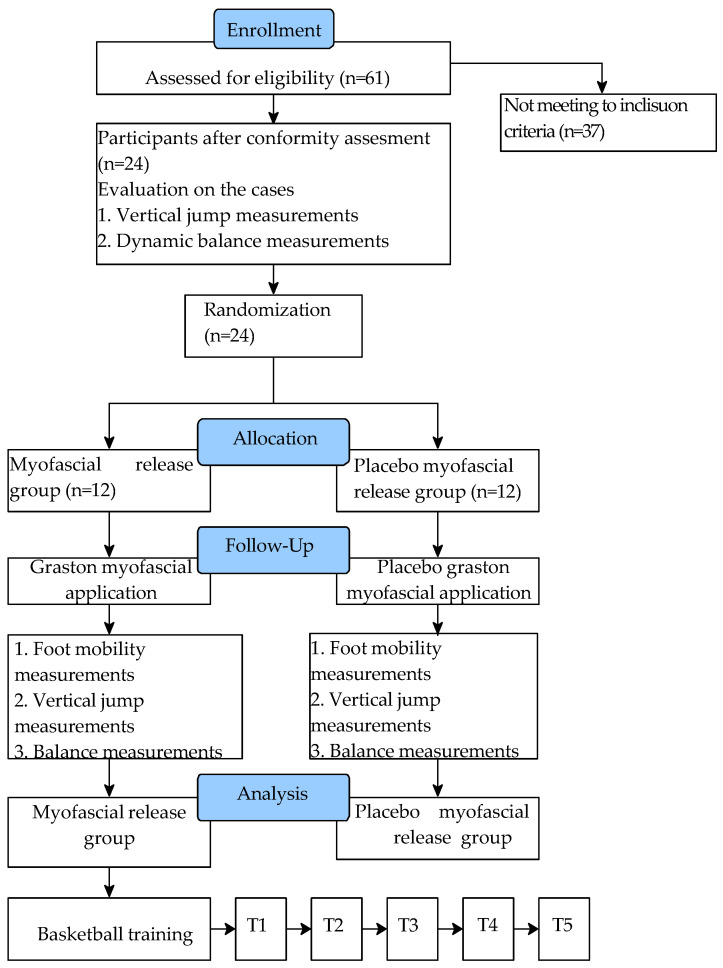
Consort diagram showing participant flow throughout the study.

The inclusion criteria included hypomobility in at least one foot of the participant. Therefore, firstly, the degree of foot mobility of all participants was measured, and the height, body weight, balance characteristics, and vertical jump levels of the participants who met the inclusion criteria were determined. Subsequently, 24 participants were randomly assigned to one of two groups, myofascial release (MR) or placebo myofascial release (PMR), in a 1:1 ratio using online randomization software (www.randomizer.org, accessed on 4 April 2024) by an independent researcher. Randomization continued until the groups achieved a statistically homogeneous structure in terms of foot hypomobility and performance characteristics before the intervention (Table 3). The MR group received myofascial release, and the PMR group received placebo (superficial) myofascial release. All myofascial release applications were performed by an expert physiotherapist. After application, the degree of foot mobility, balance characteristics and vertical jump levels of the participants were measured again. In order to determine the duration of the acute effects of mobility after myofascial release, the MR group was subjected to a 2 h standard basketball training session consisting of warm-up, dribbling, shooting, passing exercises and cooling down phases after the second measurements. Foot mobility was measured three more times at one-hour intervals in the first hour of the training, at the end of the training, and then during rest. In the fifth measurement after application, mobility measurements were completed as a result of the statistical observation that the acute effects of the application disappeared. All foot mobility and performance measurements were performed by the same researcher (Figure 1). Additionally, clinically validated and reliable technology-assisted methods and devices were utilized in all measurements in this study.

To enhance the reliability of the study and minimize biases, blinding procedures were applied to all participants and evaluators. For blinding purposes, the MR group was coded with the letter A, and the PMR group was coded with the letter B. Participants were not informed about which group they were assigned to, and all participants received the same information regarding the interventions. All release applications were performed by the same expert physiotherapist. Foot mobility and performance measurements were conducted by a separate specialist who was not informed about which participant received which intervention. Additionally, the data were analyzed using group codes rather than group names. The blinding procedures were terminated during the follow-up of the continuity of the acute effect.

#### 2.3.1. Height and Body Weight Measurements

The height measurements of the participants were measured with a wall-mounted Holtain stadiometer, and body weight and body mass index (BMI) measurements were measured with a Tanita MC-580 body analyzer.

#### 2.3.2. Warm-Up

Participants were given 15 min of active warm-up consisting of 5 min of running, 5 min of short fast ascents, and 5 min of stretching movements before the measurements.

#### 2.3.3. Evaluation of Foot Mobility

**Figure 2:**
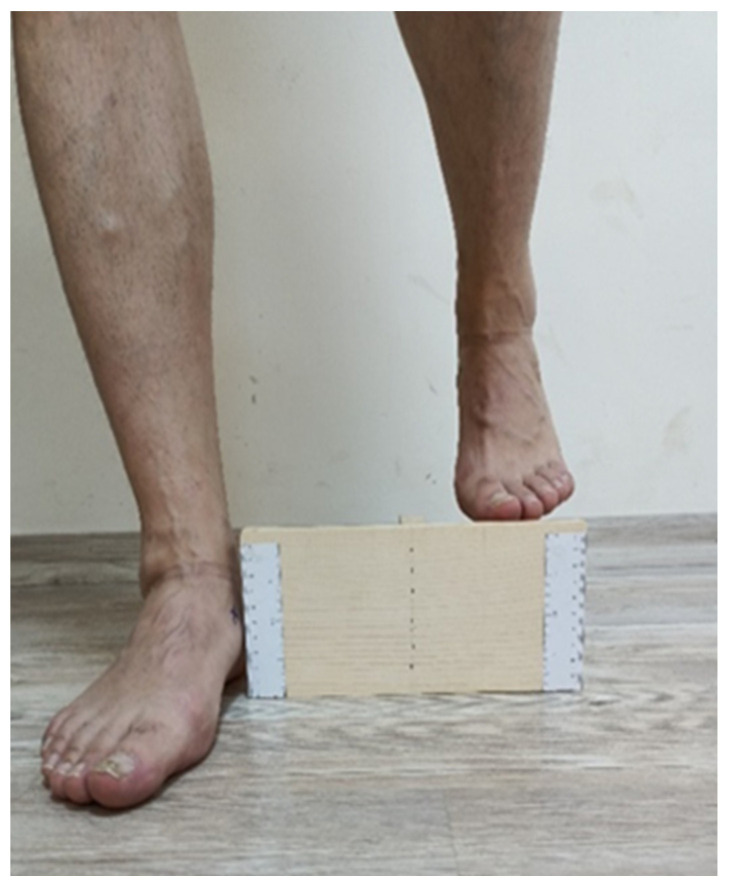
Evaluation of foot mobility.

The modified navicular drop (MND) test was used to assess foot mobility (Figure 2). The MND test is an easy, valid and clinical method used in the evaluation of foot mobility. In the navicular drop test, the navicular process was palpated externally while the subject was sitting on a chair with their foot in neutral position and the height above the ground was measured. The subject was then made to stand up and place one foot on a specially designed box that was 10.16 cm (4 inch) high without weight. The height above the ground in the navicular process for the loaded foot was measured again. Feet with a difference of less than 4 mm between unweighted and weighted measurements were recorded as hypomobile. The reference values of navicular mobility in the MND test are 0–4 mm for a hypomobile foot, 5–9 mm for a neutral foot, and 10 mm or more for a hypermobile foot [1]. The distribution of the participants’ foot hypomobility after the assessment of foot mobility as bilateral and unilateral is shown in Table 2.

#### 2.3.4. Vertical Jump Measurements

Vertical jump values were measured with an arm swing countermovement jump technique using a smart speed brand electronic jump mat. Each athlete positioned himself on the jumping mat with feet one shoulder-width apart. Then, when he felt ready, he stretched downward and jumped vertically to the highest point he could jump and fell back on the mat. Three attempts were made in the correct position; a 3 s rest time was allowed between each jump; and the best height was recorded in cm. The jumps were repeated with the knees flexed while the athlete remained in the air [21,22].

#### 2.3.5. Determination of Balance Levels

**Figure 3:**
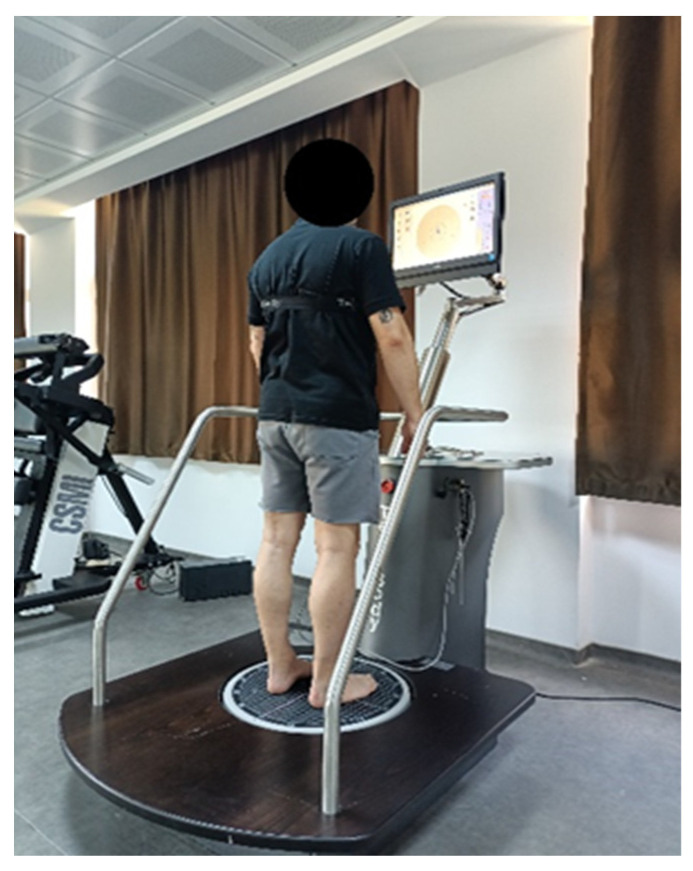
Isokinetic balance system.

A CSMI TecnoBody PK-252 isokinetic balance system measuring device was used to determine the balance levels of the participants (Figure 3). Balance measurements were made statically and dynamically. In this system, it is interpreted as an improvement in balance levels when the balance scores approach zero (0) and a worsening in balance levels when they move away from zero [23].

##### Static Balance Measurement

In the static balance measurement, the device was first calibrated, and the system was introduced to the volunteers. The “Static Stability Assessment” module of the device was selected, and the volunteers’ feet were placed on the platform with reference to the x and y lines on the platform. In the measuring position, their hands were drooping and their feet were bare. Measurements were taken over 30 s with a bipedal and eyes open. The balance score was calculated by summing the standard deviation of the front-to-back swing and the standard deviation of the right-to-left swing. An increase in the static balance score indicates that the deviations increase and the level of static balance weakens [23].

##### Dynamic Balance Measurement

The multiaxial proprioceptive assessment module of the isokinetic measurement system was used to determine the dynamic balance levels. First, the device was calibrated and the system was introduced to the volunteers. The volunteers’ feet were placed on the platform with reference to the x and y lines on the platform. In the measuring position, their hands were drooping and their feet were bare. Measurements were made with a bipedal and 10 difficulty levels for 60 s. The test was stopped and restarted when situations such as falling or touching any part of the device occurred during the measurement. Dynamic balance levels were assessed using the average tracking error (ATE). The resulting values indicate the extent to which the participant deviates or exceeds the limits of the path the participant should follow. Higher ATE scores indicate poorer dynamic balance, while lower ATE scores indicate better dynamic balance [23].

#### 2.3.6. Myofascial Release Application

The Graston massage technique was used during application. During application, the participant was placed in a prone position, and their feet were left slightly outside the massage table. The application was performed on the entire plantar surface between the calcaneal tuberosity and metatarsophalangeal joints in the form of multidirectional strokes at 30–60 degrees with the GT 4 Graston tool. The total application took an average of 5 min for each foot. An average of 60–70 strokes were performed per minute, and 10 s of rest was given every minute. The application was performed in a single session by an expert physiotherapist [18,24]. In the placebo application, unlike the actual treatment, only the posterior foot was targeted with light pressure and slow strokes close to the level of touch.

### 2.4. Statistical Analysis

The SPSS 25.0 package program was used for data analysis. The data were first subjected to the Shapiro–Wilk normal distribution test, and it was seen that the data showed a normal distribution. An independent-sample *t*-test was used to determine the differences between the groups, and a paired *t*-test was used to determine the differences before and after application. The paired *t*-test results were interpreted according to Cohen’s d effect size [25]. A repeated-measures ANOVA test was used to evaluate the effect of the acute duration of the application on foot mobility. Since the sphericity assumption was not met for both feet in the ANOVA test, the results were interpreted according to Greenhouse–Geisser, and it was seen that there were differences between times (*p* < 0.001). Effect size was interpreted using partial η^2^ (0.01 ≤ pη^2^ < 0.06 as small, 0.06 ≤ pη^2^ < 0.14 as medium, pη^2^ ≥ 0.14 as a large effect). A Bonferroni multiple comparison test was used to determine the time intervals of the acute effect. The results were analyzed at the *p* < 0.05 significance level.

## 3. Results

In Table 3, the differences between the groups before the application were analyzed, and it was seen that there was no difference between the groups in any parameter.

Table 4 shows the differences before and after the application. In the MR group, there were significant differences in the RFM [t(22) = −11.406; d = 4; *p* < 0.001], LFM [t(22) = −10.307; d = 6; *p* < 0.001], VJ [t(22) = −6.282; d = 1.13; *p* < 0.001] and DB [t(22) = 5.703; d = 1.03; *p* < 0.001] parameters. No difference was found in the SB parameter [t(22) = 1.727; *p* > 0.05]. In the PM group, no significant difference was observed in any parameter after application (*p* > 0.05).

Table 5 shows that there were significant differences between repeated measurements in the right foot [F(1.603–17.633) = 91.300; pη^2^ = 0.964; *p* < 0.001] and left foot [F(1.565–17.214) = 79.837; pη^2^ = 0.879; *p* < 0.001)] (*p* > 0.05).

When the acute effects of the application on foot mobility were evaluated according to time intervals, it was observed that there was a significant difference between Pre-m and Ipost-m (7.58 ± 1.08; *p* < 0.001), Pre-m and T1 (7.25 ± 1.13; *p* < 0.001), and Pre-m and T2 (5.92 ± 0.99; *p* < 0.001) in the right foot, and the acute effect lasted approximately 2 h. In the left foot, there was a significant difference between Pre-m (1.83 ± 1.47) and Ipost-m1 (7.08 ± 1.24; *p* < 0.001), Pre-m and T1 (6.33 ± 1.15; *p* < 0.001), Pre-m and T2 (5.17 ± 1.11; *p* < 0.001), Pre-m and T3 (3.42 ± 1.24; *p* < 0.05), and Pre-m and T4 (2.50 ± 1.45; *p* < 0.05), and the acute effect lasted approximately 4 h.

## 4. Discussion

This study examined the acute effects of myofascial release applied to the plantar sole on foot mobility and performance in basketball players with hypomobility in at least one foot. To the best of our knowledge, this is the first report in this field, and this aspect reveals the originality of the study. The results of this study showed that there was a significant positive difference in the RFM, LFM, VJ and DB parameters after application in the MR group (*p* < 0.001), while there was no difference in SB (*p* > 0.05). In the PMR group, no change was found in the parameters after placebo application (*p* > 0.05).

**Figure 4:**
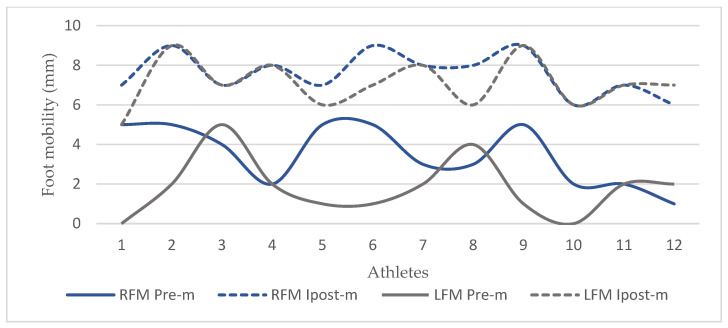
Foot mobility in MR group. Abbreviations: RFM Pre-m, right foot mobility before application; RFM Ipost-m, right foot mobility immediately after application; LFM Pre-m, left foot mobility before application; LFM Ipost-m, left foot mobility immediately after application; mm, millimeter.

**Figure 5:**
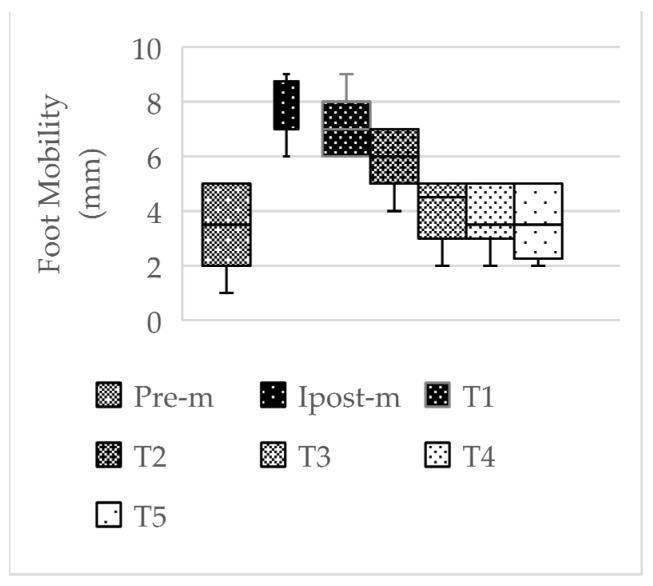
Right foot acute effect in MR group. Abbreviations: Pre-m, before application; Ipost-m, immediately after application; T1, 1 h after application; T2, 2 h after application; T3, 3 h after application; T4, 4 h after application; T5, 5 h after application; mm, millimeter.

**Figure 6:**
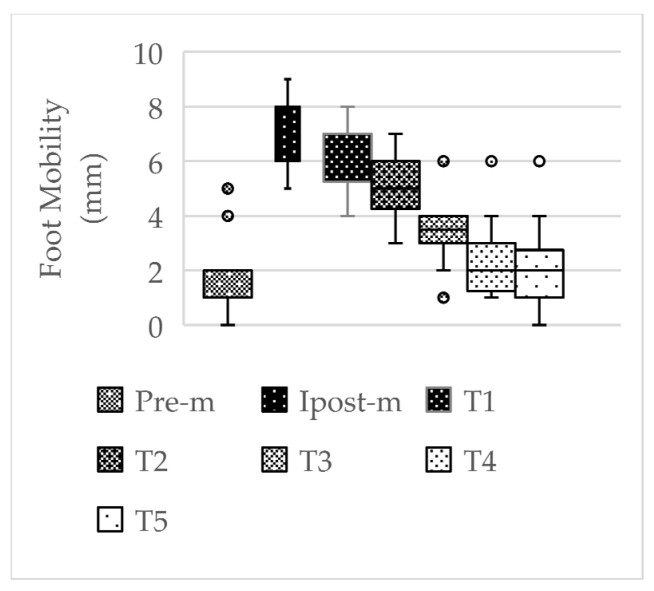
Left foot acute effect in MR group. Abbreviations: Pre-m, before application; Ipost-m, immediately after application; T1, 1 h after application; T2, 2 h after application; T3, 3 h after application; T4, 4 h after application; T5, 5 h after application; mm, millimeter.

In the literature, navicular mobility below 4 mm is considered a hypomobile foot, and 4 mm–9 mm navicular mobility is considered a normal foot [1]. In this study, the mean values of foot mobility before the application were found to be 3.50 ± 1.51 mm for RFM and 1.83 ± 1.47 mm for LFM in the MR group and 3.17 ± 2.44 mm for RFM and 2.67 ± 2.31 mm for LFM in the PMR group. In the first measurements made immediately after the application, it was determined that the mean values increased to 7.58 ± 1.08 mm for RFM and 7.08 ± 1.24 mm for LFM in the MR group. It was observed that this increase after the application was within the range of reference values and significant in both feet (Table 4, Figure 4, *p* < 0.05). It was also determined that the acute effects of the application on foot mobility lasted at least 2 h for RFM and at least 4 h for LFM after the application (Table 6, Figure 5 and Figure 6, *p* < 0.05). A standard basketball match or training session lasts approximately two hours. In a basketball game or training session, where a series of coordinative skills need to be demonstrated efficiently under a high level of intensity and quick pace, this temporary improvement in foot functionality lasting at least 2 h can be considered significant for enhancing athletic performance and reducing the risks of potential injuries.

The results of this study suggest that myofascial release produces short-term positive effects on the elimination of movement limitations in the foot. In the literature, no research in which myofascial techniques were applied to increase foot mobility in athletes with hypomobile feet was found. However, in previous studies, it has been reported that the functionality of the foot is affected by the anatomical and mechanical properties of the soft tissues in the plantar sole of the foot, and manipulative interventions on soft tissues increase the functionality of the foot [14]. Due to the direct relationship between the structural properties of soft tissues and foot mobility, the tension and stiffness of elastic structures such as muscle tendons and fascia can hinder the optimal movement of the segments that make up the foot. Myofascial release interventions reduce this tension in the soft tissues while enhancing their elasticity and flexibility, thereby increasing the range of motion in the joints. These interventions contribute to joint mobility through changes induced in the nervous system. Specifically, pressure on muscle spindles and Golgi tendon organs triggers these sensors to send signals aimed at reducing muscle tone. This neural mechanism helps the muscles relax and become less tense. Additionally, myofascial release techniques increase blood and lymphatic circulation. Enhanced circulation allows more oxygen and nutrients to reach the tissues, which in turn improves the flexibility of muscles and fascia [14,16]. The Graston technique is also recommended for myofascial release applications aimed at restoring functionality in the foot. This is because the Graston technique allows for deeper and more precise interventions in muscle and fascia tissue compared to other techniques, effectively identifying and resolving micro-adhesions within the tissue structure [18,26]. In this study, the Graston technique was preferred as a myofascial release technique in line with the recommendations of the relevant researchers and in order to maximize the mobilization effects of the technique on soft tissues. Our findings show that the approach applied in this research has effective results.

**Figure 7:**
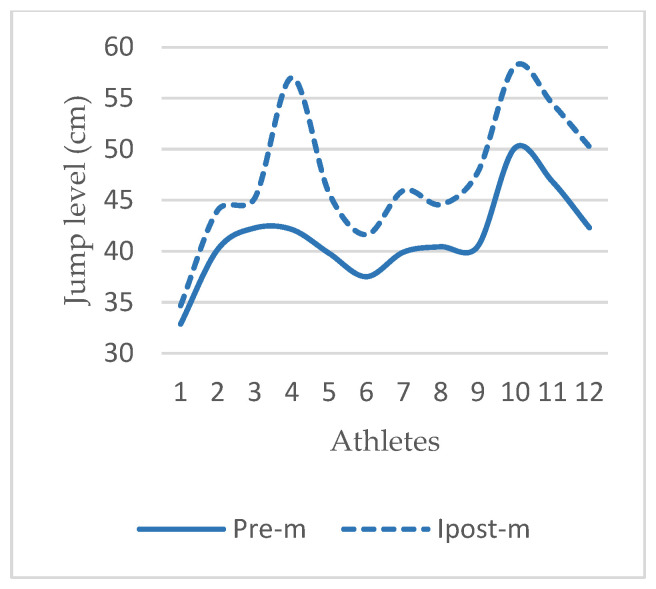
Vertical Jump (VJ) in MR group. Abbreviations: Pre-m, before application; Ipost-m, immediately after application; VJ, vertical jump; cm, centimeter; DB, dynamic balance; ATE, average tracking error.

Vertical jump, which is one of the performance elements in which the acute effects of the application were examined in this study, is a very important factor affecting success by providing an advantage to the athlete in achieving rebounds in offense and defense in basketball, blocking against shots, and performing shots such as dunks [10,27]. In the research findings, it was observed that there was a significant positive increase in the VJ levels of the athletes after the application (Table 3, Figure 7, *p* < 0.001). The foot is the main region where the necessary propulsion force is generated during jumping. Therefore, stabilization of the joints involved in the anatomical structure of the foot, neuromuscular compliance, the structure of the intrinsic flexor muscles in the sole of the foot, the medial longitudinal arch (MLA) and the plantar fascia are important factors affecting jump performance [2,3]. The medial longitudinal arch is supported by the intrinsic flexors and plantar fascia while performing energy storage and release during dynamic movements. For these reasons, the functionality of this mechanism allows for more force production and successful jumping during the propulsion phase [28]. Restrictions in the soft tissues of the foot negatively affect MLA mobilization and stabilization [29,30]. The increase in VJ levels in the current study is thought to be due to the contribution of the application to the plantar sole to foot functionality.

**Figure 8:**
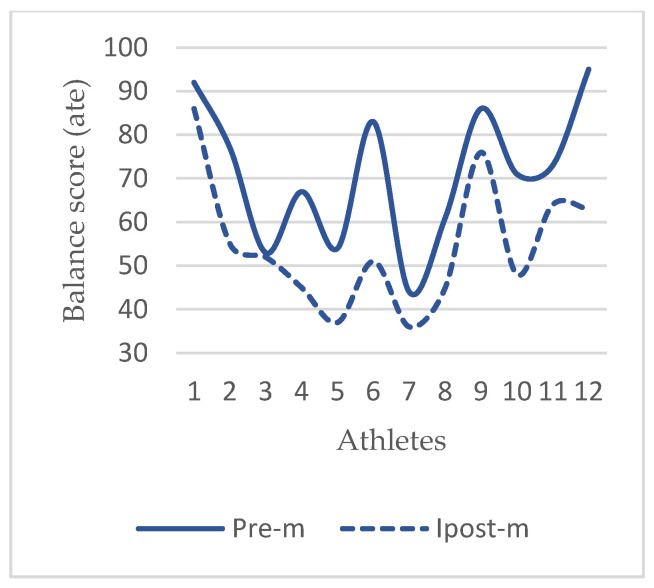
Dynamic balance (DB) in MR group. Abbreviations: Pre-m, before application; Ipost-m, immediately after application; VJ, vertical jump; cm, centimeter; DB, dynamic balance; ATE, average tracking error.

In this study, one of the performance elements in which the acute effects of the practice were examined was balance ability. Basketball is a sport that constantly requires fast and sudden movements. Athletes need improved balance ability in order to be successful in fast and sudden movements and to be protected from injuries at the highest level [9]. In the results of this study, it was observed that there was a significant improvement in the DB skills of the athletes after the application (Table 3, Figure 8, *p* < 0.001). The functionality of the foot comes into play in absorbing the load applied at the moment of movement, transferring it to the ground properly, stabilizing the center of mass and maintaining posture against gravity [11]. In previous studies, it has been reported that stiff plantar fascia worsens balance performance by reducing mobility [7], an increase in MLA stiffness may impair dynamic balance [8], and an increase in MLA height is associated with decreased mediolateral control in a single-limb stance [31]. In one study, it was found that a single session of myofascial induction applied to the plantar surface of the foot increased the forefoot maximum pressure and forefoot ground contact surface in individuals with plantar fascia restriction [32]. In another study, a positive relationship between a wide forefoot and balance performance was reported [33], and in another study conducted, it was reported that increased forefoot varus may negatively affect postural stability [34]. Related studies show that changes in the anatomical structure of the foot and interventions to the structure affect balance mechanisms. From this point of view, the results of these related studies are similar to the results of this study. Additionally, the plantar surface of the foot is an area densely populated with mechanoreceptors that send information about the body’s position to the central nervous system, affecting proprioception. Proprioception plays a critical role in postural balance and motor function in athletes, as well as in the general population and individuals with neurological disorders. An increase in the feedback activation of plantar mechanoreceptors enhances proprioception and thus improves balance [35,36]. In the literature, it is emphasized by researchers that myofascial release techniques are effective in increasing proprioception [37,38]. In this study, it is thought that the improvement seen in DB levels after application is due to the increase in foot functionality and proprioceptive feedback.

Another result of this study was that there was no significant difference in SB skills after the application (Table 4, *p* > 0.05). In static balance, on a much more stable surface compared to dynamic balance, the support base is maintained with minimal movement, while in dynamic balance, on a moving surface, the external forces acting on the body are countered by the muscles and soft tissues around the joints. Additionally, different conditions and interventions may affect dynamic balance while not affecting static balance [39,40]. It is thought that the lack of significant improvement in static balance after the application in the study is due to the fact that joint mobility is used much less when maintaining balance on a motionless surface. Nevertheless, further research is needed to make a definitive judgment.

### Limitations

The first limitation of this study is that the application acutely removes hypomobility in the foot, and its short-term effects are known. While the short-term results are promising, it is not known how hypomobility will be affected in the long term. Furthermore, the absence of a long-term follow-up complicates the assessment of the lasting benefits of the intervention. The second limitation of this study is that the sample size consists of 12 participants in each group. Although the number of participants is considered sufficient for power analysis, having a larger sample size in similar studies could enhance the strength of the research. The third limitation of this study was the exclusion of bilateral or unilateral differences in the evaluation of foot hypomobility. This is because the groups were homogeneous in terms of mobility and performance characteristics before Graston application, and the sample size would be insufficient when divided in this way. The fourth limitation of this study is the difficulty of blinding in relation to the potential placebo effects of the intervention. Due to the nature of manual therapy, participants physically feel the treatment, which may lead them to engage in behaviors that could affect performance changes. Additionally, while participants may not know which group they belong to, blinding is nearly impossible from the practitioners’ perspective. This is particularly concerning in studies involving less experienced practitioners, as their knowledge of the intervention group could lead to behavioral changes. The fifth limitation of this study is the inability to account for confounding factors such as the time of day, social life and nutrition. While the reliability of the study was enhanced by having all interventions conducted by the same expert physiotherapist, this also resulted in other confounding factors not being considered. Factors such as the time of day, social life and nutrition can influence the results and may restrict generalizability. However, those who had participated in any high-intensity sport activity in the last 24 h were excluded from this study to prevent confounding factors such as fatigue and previous activity levels. The last limitation of this study is the restrictions in epidemiological research regarding the prevalence of foot hypomobility in basketball players and other athletes. In sports like basketball, where vertical and horizontal jumps are fundamental to high athletic performance, years of intensive training and repetitive injuries can lead to restrictions in foot mobility. However, gaps in the literature reduce the effectiveness of potential solutions that can be proposed for foot hypomobility.

## 5. Conclusions

Without making definitive judgments, the results of this study indicate that a single session of myofascial release applied to the plantar sole temporarily increased foot mobility and improved vertical jump and dynamic balance performance in basketball players with chronic hypomobility. These results may be promising in terms of Garston myofascial release being a drug-free and non-invasive option for the acute relief of foot hypomobility in athletes, contributing to performance. Nevertheless, there is a need for more evidence to explain the underlying mechanisms behind these findings. It is thought that increasing research on foot hypomobility, hypomobility-related problems and compensatory methods in athletes will contribute to the field.

Practical implications:

- Foot mobility increased after the application. This technique may be beneficial in restoring mobility and functionality to the foot.
- Improvements were observed in vertical jump and dynamic balance levels after the application. The application may be beneficial for enhancing performance.
- The acute effect on foot mobility was observed to last at least 2 h. The application may be recommended before training and competitions.
- These results can be considered by coaches and athletes in female basketball and other sports with similar physical fitness parameters.

## Figures and Tables

**Table 1:** The demographic characteristics of the participants.

Variable	MR Group ($\bar{x}$ ± sd)	PMR Group ($\bar{x}$ ± sd)	t-Value	*p*-Value *
Age (years)	24.08 ± 3.06	22.83 ± 2.52	1.093	0.286
Weight (kg)	86.83 ± 13.79	82.17 ± 6.47	1.061	0.300
Height (cm)	187.08 ± 6.20	186.00 ± 6.20	0.427	0.673
BMI (kg/cm^2^)	24.10 ± 0.77	23.54 ± 0.90	1.664	0.110

Abbreviations: MR, myofascial release; PMR, placebo myofascial release; $\bar{x}$, mean; sd, standard deviation; kg, kilograms; cm, centimeters; BMI, body mass index; *p* < 0.05 was considered statistically significant. *p*-values are from an independent *t*-test *.

**Table 2:** Distribution of participants’ foot hypomobility as bilateral and unilateral.

Variable	HRF	HLF	HBF
AP	3	8	13
MR group	1	5	6
PMR group	2	3	7

Abbreviations: HRF, hypomobility in the right foot; HLF, hypomobility in the left foot; HBF, hypomobility in both feet; APs, all participants; MR, myofascial release; PMR, placebo myofascial release.

**Table 3:** Differences between groups before application.

Variable	MR Group ($\bar{x}$ ± sd)	PMR Group ($\bar{x}$ ± sd)	t-Value	*p*-Value *
RFM (mm)	3.50 ± 1.51	3.17 ± 2.44	0.402	0.691
LFM (mm)	1.83 ± 1.47	2.67 ± 2.31	−0.910	0.372
VJ (cm)	41.25 ± 4.31	42.62 ± 6.32	−0.619	0.542
SB (bs)	7.08 ± 2.71	6.75 ± 1.48	0.373	0.713
DB (ate)	71.33 ± 16.19	60.17 ± 14.60	1.775	0.090

Abbreviations: MR, myofascial release; PMR, placebo myofascial release; $\bar{x}$, mean; sd, standard deviation; RFM, right foot mobility; mm, millimeters; LFM, left foot mobility; VJ, vertical jump; cm, centimeter; SB, static balance; bs, balance score; DB, dynamic balance; ATE, average tracking error. *p* < 0.05 was considered statistically significant. *p*-values are from an independent *t*-test *.

**Table 4:** Differences before and after application.

Variable	Groups	Pre-m ($\bar{x}$ ± sd)	Ipost-m ($\bar{x}$ ± sd)	t-Value	*p*-Value *	d-Value
RFM (mm)	MR group	3.50 ± 1.51	7.58 ± 1.08	−11.406	0.001 *	4
	PMR group	3.17 ± 2.44	3.25 ± 2.53	−1.000	0.339	-
LFM (mm)	MR group	1.83 ± 1.47	7.08 ± 1.24	−10.307	0.001 *	6
	PMR group	2.67 ± 2.31	2.83 ± 2.25	−1.483	0.166	-
VJ (cm)	MR group	41.25 ± 4.31	47.45 ± 6.68	−6.282	0.001 *	1.13
	PMR group	42.62 ± 6.32	43.13 ± 5.50	−1.227	0.246	-
SB (bs)	MR group	7.08 ± 2.71	5.75 ± 1.29	1.727	0.112	-
	PMR group	6.75 ± 1.48	6.25 ± 1.96	0.838	0.420	-
DB (ate)	MR group	71.33 ± 16.19	54.83 ± 15.10	5.703	0.001 *	1.03
	PMR group	60.17 ± 14.60	63.25 ± 11.43	−0.828	0.425	-

Abbreviations: Pre-m, before application; Ipost-m, immediately after application; MR, myofascial release; PMR, placebo myofascial release; $\bar{x}$, mean; sd, standard deviation; RFM, right foot mobility; mm, millimeter; LFM, left foot mobility; VJ, vertical jump; cm, centimeter; SB, static balance; bs, balance score; DB, dynamic balance; ATE, average tracking error. *p* < 0.05 was considered statistically significant. *p*-values are from a paired *t*-test *.

**Table 5:** Examination of the acute effects of the application on foot mobility.

Variable	Source	Sum of Squares	Df	Mean Squares	F	*p*-Value *	pη^2^
RFM	Difference between measurements	229.238	1.603	143.004	91.300	0.001 *	0.964
	Error	27.619	17.633	1.566			
LFM	Difference between measurements	326.952	1.565	208.923	79.837	0.001 *	0.879
	Error	45.048	17.214	2.617			

Abbreviations: RFM, right foot mobility; LFM, left foot mobility; pη^2^, partial η^2^. *p* < 0.05 was considered statistically significant. *p*-values are from a repeated-measures ANOVA test *.

**Table 6:** Evaluation of the acute effects of the application according to time intervals.

Variable	Pre-m ($\bar{x}$ ± sd)	Ipost-m ($\bar{x}$ ± sd)	T1 ($\bar{x}$ ± sd)	T2 ($\bar{x}$ ± sd)	T3 ($\bar{x}$ ± sd)	T4 ($\bar{x}$ ± sd)	T5 ($\bar{x}$ ± sd)
RFM (mm)	3.50 ± 1.51	7.58 ± 1.08 *	7.25 ± 1.13 *	5.92 ± 0.99 *	4 ± 1.13	3.75 ± 1.21	3.67 ± 1.30
LFM (mm)	1.83 ± 1.47	7.08 ± 1.24 *	6.33 ± 1.15 *	5.17 ± 1.11 *	3.42 ± 1.24 *	2.50 ± 1.45 *	2.08 ± 1.68

Abbreviations: Pre-m, before application; $\bar{x}$, mean; sd, standard deviation; Ipost-m, immediately after application; T1, 1 h after application; T2, 2 h after application; T3, 3 h after application; T4, 4 h after application; T5, 5 h after application; mm, millimeter; RFM, right foot mobility; LFM, left foot mobility. *p* < 0.05 was considered statistically significant. *p*-values are from a Bonferroni pairwise comparison test *.

## Data Availability

The data presented in this study are available upon request from the corresponding author.
